# A Simplified Screening Model to Predict the Risk of Gestational Diabetes Mellitus in Caucasian and Latin American Pregnant Women

**DOI:** 10.3390/genes15040482

**Published:** 2024-04-11

**Authors:** María Arnoriaga-Rodríguez, Irene Serrano, Mateo Paz, Ana Barabash, Johanna Valerio, Laura del Valle, Rocio O’Connors, Verónica Melero, Paz de Miguel, Ángel Diaz, Cristina Familiar, Inmaculada Moraga, Mario Pazos-Guerra, Mercedes Martínez-Novillo, Miguel A. Rubio, Clara Marcuello, Ana Ramos-Leví, Pilar Matia-Martín, Alfonso L. Calle-Pascual

**Affiliations:** 1 Endocrinology and Nutrition Department, Hospital Clínico Universitario San Carlos, Instituto de Investigación Sanitaria del Hospital Clínico San Carlos (IdISSC), 28040 Madrid, Spain; maria.arnoriaga.rodriguez@gmail.com (M.A.-R.); ana.barabash@gmail.com (A.B.); valeriojohanna@gmail.com (J.V.); lauradel_valle@hotmail.com (L.d.V.); veronica.meleroalvarez10@gmail.com (V.M.); pazdemiguelnovoa@gmail.com (P.d.M.); joseangel.diaz@salud.madrid.org (Á.D.); cristinafamiliarcasado@gmail.com (C.F.); inmaculada.moraga@salud.madrid.org (I.M.); mario_pazos_guerra@hotmail.com (M.P.-G.); marubioh@gmail.com (M.A.R.); clara994@hotmail.com (C.M.); ana_ramoslevi@hotmail.com (A.R.-L.); 2 Unidad de Apoyo a la Investigación, Instituto de Investigación Sanitaria del Hospital Clínico San Carlos (IdISSC), Biomedical Research Networking Center in Cancer (CIBERONC), 28040 Madrid, Spain; isgarcia@salud.madrid.org (I.S.); mateo.paz@salud.madrid.org (M.P.); 3 Facultad de Medicina, Medicina II Department, Universidad Complutense de Madrid, 28040 Madrid, Spain; 4 Centro de Investigación Biomédica en Red de Diabetes y Enfermedades Metabólicas Asociadas (CIBERDEM), 28029 Madrid, Spain; 5 Clinical Laboratory Department, Hospital Clínico Universitario San Carlos, Instituto de Investigación Sanitaria del Hospital Clínico San Carlos (IdISSC), 28040 Madrid, Spain; mercedes.martineznovillo@salud.madrid.org

**Keywords:** gestational diabetes mellitus, Caucasian, Latin American, women, age, body mass index, fasting plasma glucose, single-nucleotide polymorphisms

## Abstract

The pathophysiology of gestational diabetes mellitus (GDM) comprises clinical and genetic factors. In fact, GDM is associated with several single nucleotide polymorphisms (SNPs). This study aimed to build a prediction model of GDM combining clinical and genetic risk factors. A total of 1588 pregnant women from the San Carlos Cohort participated in the present study, including 1069 (67.3%) Caucasian (CAU) and 519 (32.7%) Latin American (LAT) individuals, and 255 (16.1%) had GDM. The incidence of GDM was similar in both groups (16.1% CAU and 16.0% LAT). Genotyping was performed via IPLEX Mass ARRAY PCR, selecting 110 SNPs based on literature references. SNPs showing the strongest likelihood of developing GDM were rs10830963, rs7651090, and rs1371614 in CAU and rs1387153 and rs9368222 in LAT. Clinical variables, including age, pre-pregnancy body mass index, and fasting plasma glucose (FPG) at 12 gestational weeks, predicted the risk of GDM (AUC 0.648, 95% CI 0.601–0.695 in CAU; AUC 0.688, 95% CI 0.628–9.748 in LAT), and adding SNPs modestly improved prediction (AUC 0.722, 95%CI 0.680–0.764 in CAU; AUC 0.769, 95% CI 0.711–0.826 in LAT). In conclusion, adding genetic variants enhanced the prediction model of GDM risk in CAU and LAT pregnant women.

## 1. Introduction

Gestational diabetes mellitus (GDM) is defined as any degree of glucose intolerance with onset or first recognition during pregnancy [1]. It is considered one of the most common complications of this period with its prevalence depending on the population studied and the diagnostic criteria used. A recent meta-analysis provided a global standardized prevalence of 14.2% in women between 25 and 30 years using the universal oral glucose tolerance test (OGTT) and the International Association of the Diabetes and Pregnancy Study Groups (IADPSG) diagnostic criteria [2]. Unfortunately, figures continue to rise due to increased maternal obesity and advanced age at childbearing [3].

GDM confers maternal, fetal, and neonatal adverse outcomes [4] and also increases the risk of type 2 diabetes (T2DM), obesity, and cardiometabolic disease in the mother and the offspring later in life [5,6,7]. In fact, women with GDM have a nearly 10-fold higher risk of developing T2DM compared to women with normoglycemia during pregnancy [7]. Therefore, pregnancy is a crucial period for decreasing both perinatal and long-term medical complications.

Environmental factors interact with genetics in the development of GDM. Previous GDM, family history of diabetes, ethnicity, parity, and higher maternal age and body mass index are the strongest clinical predictors for GDM [8]. The role of genetics in GDM remains poorly defined [3] and needs to be further explored. Genome-wide association studies (GWASs) have identified single-nucleotide polymorphisms (SNPs) associated with GDM [9,10]. SPNs from different genes such as *TCF7L2*, *GCK*, *KCNJ11*, *IGF2BP2*, *IRS1* [11], *MTNR1B*, and *CDKAL1* [10,11] have been linked to higher GDM risks. In addition, some studies evaluated genetic data through genetic risk scores (GRSs) [12]. However, their predictive value is sometimes limited, and other indicators might be combined to enhance results.

Therefore, the aim of the present study is to build a prediction model of GDM based on a combination of genetic, clinical, and demographic information. We hypothesized that genetics in combination with well-known environmental risk factors might improve the prediction of GDM in pregnant women and, thus, short- and long-term medical outcomes. Identifying women at risk as early as possible is the first step to designing preventive strategies.

## 2. Methods

### 2.1. Population

The Hospital Clínico San Carlos is a public hospital located in the central area of Madrid, with a healthcare population of 445,000 inhabitants assigned during the performance of this study between 2015 and 2017. Prenatal screening consultation is located in the hospital where all pregnant women were admitted between gestational weeks (GW) 9 and 12, and the first ultrasound and screening tests were carried out to detect the risk of chromosomal alterations.

San Carlos Cohort: The San Carlos Cohort is constituted of women who took part in studies for the prevention of gestational diabetes carried out between 2015 and 2017, those who are registered in a randomized control trial (ISRCTN84389045; https://doi.org/10.1186/ISRCTN84389045, accessed on 29 January 2024) [13], and those registered in a prospective real-world study (ISRCTN13389832; https://doi.org/10.1186/ISRCTN13389832, accessed on 29 January 2024) [14]. The studies were approved by the Clinical Trials Committee of the Hospital Clínico San Carlos (CI13/296-E, CI16/442-E and CI16/316). All women signed the informed consent form.

A total of 3036 women were enrolled in the studies, and 2156 women samples were obtained for genetic study. Normoglycemic (<92 mg/dL) pregnant women at 8–12 GW were invited to participate upon their first ultrasound visit. The gestational age at entry for inclusion was based on the one obtained in this first ultrasound. The inclusion criteria are as follows: ≥18 years old, single gestation, acceptance of participation in the study, and signature of the consent form. The exclusion criteria are as follows: gestational age at entry > 14 GW, intolerance to nuts or extra virgin olive oil, medical conditions, or pharmacological therapy that could compromise the effect of the intervention and/or the follow-up program.

Genotyping samples were obtained from 1711 (79.4%) women, of which 1645 (96%) samples were sufficient to determine >95 SNPs. A total of 1588 (93%) women, 1069 CAU, and 519 LAT were assessed in this study. Fifty-seven women from other minorities (China, Philippines, India, North African, and Sub-Saharan Africa) were excluded in this study because of their minimal representation.

### 2.2. Gestational Diabetes Mellitus Diagnosis

Diagnosis of GDM was performed via a 75 g OGTT at 24–28 GW using the IADPSG criteria [15].

### 2.3. Clinical and Laboratory Parameters

Clinical, demographic, and anthropometric data were collected in a face-to-face appointment and examination. FPG levels were determined via the glucose oxidase method in fresh plasma samples, and lipid profiles were measured using an analyzer (CobasR 8000 c702, Roche Diagnostics, Basel, Switzerland).

### 2.4. Lifestyle Evaluation

Dietary intake and physical activity were evaluated using the 14-point Mediterranean Diet Adherence Screener (MEDAS) [16]. This questionnaire considers 14 items and evaluates adherence to the Mediterranean Diet. The compliance of each item provides +1 points. A score of ≥10 is considered a target.

### 2.5. Genotype Analysis

DNA was isolated using the buffy coat obtained from one of the blood samples collected during the OGTT (K2-EDTA tubes). DNA concentration and purity were determined using a Nanodrop 2000 spectrophotometer (Thermo Fisher Scientific, Wilmington, DE, USA). A 16 Maxwell automated DNA extractor (Promega, Dübendorf, Switzerland) was used to extract DNA. SNP genotyping was performed using the MassARRAY system (Agena Bioscience, San Diego, CA, USA) combined with the iPLEX Gold SNP Genotyping method. PCR and iPLEX extension primers were designed using Assay Design Suite V2.0 online (Agena Bioscience, San Diego, CA, USA). The reaction products were transferred from the microplates into SpectroChipArrays with a Nanodispenser (Agena Bioscience). An Agena Bioscience MassARRAY Spectrometer was used to perform a MALDI-TOF–MS analysis. Genotyping results were analyzed via the MassArrayTyper 4.0 software. Panels were designed in collaboration with PATIA, and genotyping was performed at the Epigenetic and Genotyping Laboratory, Central Unit for Research in Medicine (UCLM), Faculty of Medicine, University of Valencia, Valencia, Spain. A comprehensive description of the genotyping methodology was previously published (https://doi.org/10.3389/fendo.2022.1036088, accessed on 29 January 2024).

### 2.6. Statistical Analyses

Qualitative variables were summarized by their number and frequencies, normally distributed continuous variables by the mean and standard deviation (±SD), and non-normally distributed continuous variables by the median and interquartile range (IQR: P_25_–P_75_).

All analyses were carried out by stratifying the sample by ethnicity, according to the two categories present in the data: Caucasian (CAU) and Latin (LAT).

Differences in clinical, laboratory, anthropometric, and genetic variables (age, pre-pregestational BMI, and FPG at 12 GW) between women with and without GDM were tested for significance using Student’s *t*-test.

Independent variables including age (≤35 and >35 years) and pre-pregnancy BMI (<25 and ≥25 kg/m^2^) were categorized based on the known categories of GDM risk. For FPG at 12 GW (≤83.5 and >83.5 mg/dL in CAU and ≤82.5 and >82.5 mg/dL in LAT, respectively), we defined an optimal cut-off threshold for our cohort by performing an ROC curve and stratifying by ethnicity. The identification of the cut-off value required the Youden Index, which maximizes the sum sensitivity and specificity.

A set of 110 SNPs with known associations with glycemic metabolism and GDM [9,11,17,18,19,20,21] were analyzed. Specifically, we selected SNPs from GCKR, TCF7L2, PPARG, and IGF-1 genes related to fasting insulin levels and SNPs from PROX1, GCKR, G6PC2, ADCY5, SLC2A2, IGF2BP2, DGKB, GCK, SLC30A8, GLIS3, CDKN2B, ADRA2A, TCF7L2, CRY2, MADD, FADS1, and MTNR1B genes linked to fasting glucose levels [17]. A detailed description of studied SNPs was previously published [22].

To evaluate the association between each SNP individually and GDM risk, univariate binary logistic regression models were used. Genetic analysis was performed using R 4.3.1.

SNPs were categorized according to the reference allele and according to the previous literature (REF). By contrast, the alternate category (ALT) included either heterozygous or homozygous mutations [22]. For each test, the corresponding p-value was obtained. SNPs previously identified [22] and/or with a *p*-value of less than 0.05 in the present study were selected for the multivariate analysis.

Finally, three multiple logistic regression analyses were performed for GDM risk: first, the multivariate completed model; second, the stepwise backward model; and third, the stepwise forward model with the 0.05 *p*-value set for entering and exclusion. The final model selected an optimal model with the best predictors together. The results of the regression models were presented using the odds ratio (OR), its 95%CI, and the *p*-value.

ROC analysis was used as a diagnostic tool for logistic regression. The ROC curve with the AUC for the probabilities predicted by the final models of each analysis and its 95% CIs were calculated and compared.

## 3. Results

### 3.1. Phenotype Data

A total of 1588 pregnant women, comprising 1069 (67.3%) Caucasian (CAU) and 519 (32.7%) Latin American (LAT) individuals, were studied. The phenotype characteristics of pregnant women at entry (<12 GW) are represented in Table 1.

GDM was diagnosed in 172 (16.1%) CAU and 83 (16.0%) LAT pregnant women. CAU and LAT pregnant women showed higher pre-pregnancy body mass index (BMI) and fasting plasma glucose (FPG) levels at 12 GW and a more prevalent history of GDM than their counterparts without GDM. LAT women with GDM exhibited an older age at pregnancy, also showing a similar trend in the CAU group (Table 1).

Based on the common differences between women with and without GDM observed in both races, age, pre-pregnancy BMI, and FPG at 12 GW were studied for the association with GDM risk. Furthermore, regarding their risk, these three variables were considered categorized as follows: age ≤ 35 years and age > 35 years; pre-pregnancy BMI < 25 kg/m^2^ and pre-pregnancy BMI ≥ 25 kg/m^2^; FPG at 12 GW ≤ 83.5 mg/dL and >83.5 mg/dL for CAU and FPG at 12 GW ≤ 82.5 mg/dL and >82.5 mg/dL for LAT.

### 3.2. Genotype Data

For each ethnicity, univariate logistic regression was performed for the 110 SNPs and 1588 samples. The genetic variants showing significant differences between women with and without GDM and/or those previously identified in our cohort [22] are shown in Table S1 for CAU and Table S2 for LAT.

To determine if clinical variables provided better adjustments as continuous variables or categorical ones (with the calculated cut-offs), we used a stepwise selection strategy on different models. This strategy consisted of eliminating variables from the model that were found to be non-significant: the first model with the relevant SNP and quantitative age and pre-pregnancy BMI; the second model with the relevant SNP and quantitative age, pre-pregnancy BMI, and FPG at 12 GW; the third model with the relevant SNP and categorized age and pre-pregnancy BMI; the fourth model with the relevant SNP and categorized age, pre-pregnancy BMI, and FPG at 12 GW. In receiver operating curve (ROC) analyses, the area under the curve (AUC) was higher when we considered the categorized independent variable.

#### 3.2.1. Caucasian Ethnicity Findings

**Figure 1:**
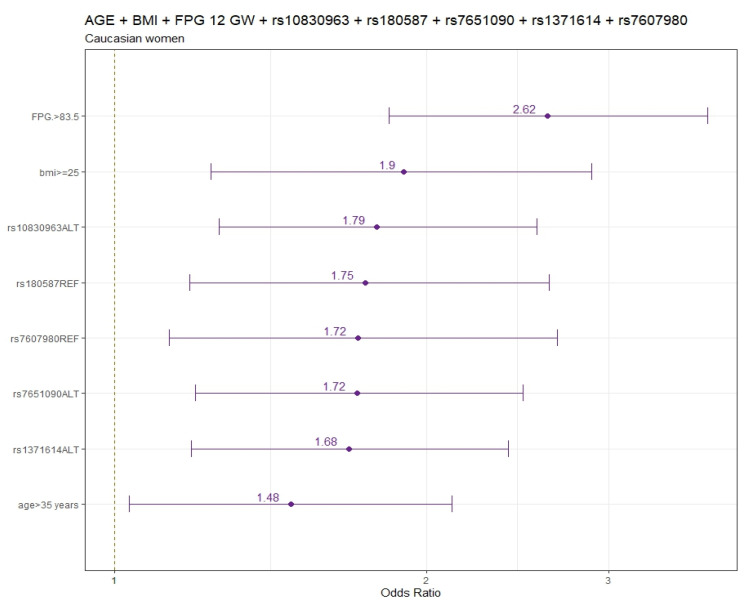
Odds ratio and 95% confidence interval for GDM in CAU. CAU, Caucasian; FPG, fasting plasma glucose; GW, gestational week.

The final models for pregnant CAU women are summarized in Figure 1.

The logistic regression models including age and pre-pregnancy BMI (Table S3) and also considering FPG 12 GW for CAU women (Table S4) are displayed in both tables. Adjusted by age and pre-pregnancy BMI, the genetic variants that were significantly associated with increased GDM risks were rs10830963 (OR:95% CI) (1.81:1.27–2.57), rs7651090 (1.73:1.21–2.49), and rs1371614 (1.66:1.18–2.35), whereas rs7607980 (0.58:0.37–0.88), rs180587 (0.59:0.40–0.87), and rs3783347 (0.67:0.45–0.98) variants were significantly associated with decreased GDM risk (Table S3).

When FPG was included in the adjusted model (age, pre-pregnancy BMI, and FPG at 12 GW), the genetic variants significantly associated with increased GDM risk were rs10830963 (1.79:1.26–2.56), rs7651090 (1.72:1.20–2.50), and rs1371614 (1.68:1.19–2.40), and the variants significantly associated with decreased GDM risk were rs180587 (0.57:0.38–0.85) and rs7607980 (0.58:0.37–0.89) (Table S4).

The receiver operating curve (ROC) analysis for the logistic regression for Caucasian women is summarized in Table 2.

In the models, the power for predicting GDM when only age was included was 0.535:0.494–0.576 (AUC:95% CI) (Figure S1a); for age and pre-pregnancy BMI, it was 0.573:0.527–0.619 (Figure S1b); for age, pre-pregnancy BMI, and FPG at 12 GW, it was 0.644:0.597–0.691 (Figure S1c). When the selected SNPs were considered, the power of prediction increased to 0.714:0.672–0.756 (Figure S1d), including age, pre-pregnancy BMI, and FPG at 12 GW.

#### 3.2.2. Latin American Ethnicity Findings

**Figure 2:**
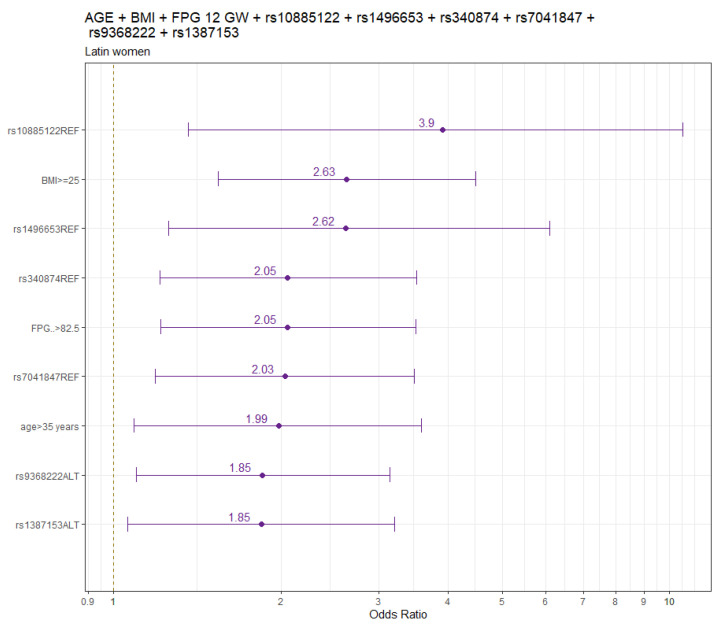
Odds ratio and 95% confidence interval for GDM in LAT. LAT, Latin American; FPG, fasting plasma glucose; GW, gestational week.

Figure 2 shows the final models’ findings for pregnant LAT women.

The logistic regression model for LAT women is summarized in Tables S5 and S6.

Adjusted by age and pre-pregnancy BMI, the genetic variants significantly associated with increased GDM risk (odds ratio:95% CI) were rs1387153 (1.88:1.09–3.21) and rs9368222 (1.77:1.06–2.95), and variants significantly associated with decreased GDM risk were rs10885122 (0.30:0.11–0.84), rs1496653 (0.46:0.21–0.91), rs7041847 (0.49:0.29–0.82), and rs340874 (0.50:0.30–0.83) (Table S5).

When FPG values at 12 GW were included in the adjusted model by age and pre-pregnancy BMI, the results did not change. The genetic variants significantly associated with increased GDM risk were rs9368222 (1.85:1.10–3.14) and rs1387153 (1.85:1.06–3.20), and the variants significantly associated with decreased GDM risk were rs10885122 (0.26:0.09–0.73), rs1496653 (0.38:0.16–0.80), rs340874 (0.49:0.29–0.82), and rs7041847 (0.49:0.29–0.84). (Table S6).

The logistic regression analysis (ROC) for LAT women is summarized in Table 3.

The power of GDM prediction including only age was 0.543:0.489–0.597 (Figure S2a); with age and pre-pregnancy BMI, it was 0.651:0.590–0.713 (Figure S2b); for age, pre-pregnancy BMI, and FPG 12 GW, it was 0.685:0.626–0.745 (Figure S2c). When we considered the selected SNPs, the power of prediction increased to 0.760:0.701–0.819, including age and pre-pregnancy BMI and adding FPG at 12 GW (Figure S2d).

## 4. Discussion

GDM is emerging as a public health concern, affecting one in six pregnancies worldwide in 2019 [23]. It not only entails maternal, fetal, and neonatal complications during pregnancy but also an increased risk of future T2DM and cardiovascular disease for both the mother and the offspring. Therefore, gestation might be a crucial period for optimal intervention and risk modification. However, some challenges must be overcome to translate this beneficial management into clinical practice. One of these challenges is the correct identification of GDM cases [24].

Different strategies have been proposed to identify GDM, but there is no consensus on the optimal approach [24]. In our setting, all pregnant women are screened at 24 gestational weeks using a one-step strategy via a 2 h 75 g OGTT recommended by the IADPSG and endorsed by the American Diabetes Association (ADA), the World Health Organization, and the International Federation of Gynecology and Obstetrics [24,25,26,27]. Nevertheless, the prompt identification of women who are more likely to develop GDM would allow improving management and, thus, short- and long-term metabolic outcomes.

Combining well-known environmental GDM risk factors and genetic variants associated with T2DM and GDM entails a more informative strategy than the current one. In fact, the genetics of GDM are not fully understood and although some studies evaluated genetic data through genetic risk scores, other additional parameters could be further included and merged to enhance results [12].

Accordingly, we conducted a case–control study of 1588 CAU and LAT pregnant women to explore the association between GDM risk and some genetic risk score models. We identified different SNPs that are significantly associated with increased or decreased GDM risk after performing stratified logistic regression analyses adjusted by age, pre-pregnancy BMI, and FPG 12 GW, which are clinical features that are significantly different between women with and without GDM in our cohort. Then, six SNPs were selected to build the final genetic risk score models. The power of prediction improved when genetic information was included. Thus, if the risk score is applied during the first weeks of pregnancy, the AUC would increase from 0.573 and 0.644, including only age and pre-pregnancy BMI, to 0.684 and 0.714, adding the selected SNPs in CAU and LAT, respectively. In line with this, at 12 GW, the AUC would increase from 0.651 and 0.685, including only age, pre-pregnancy BMI, and FPG at 12 GW, to 0.745 in CAU, and it increased to 0.760 in LAT when adding the selected SNPs, showing the highest predictive value of GDM in the latter and in our study. Therefore, the present risk score could help identify women who are more likely to develop GDM and would allow us to initiate proper management promptly. In addition, due to the high negative predictive value, women with a lower risk of developing GDM would be ruled out.

These results are consistent with previous evidence in which well-recognized risk factors and the combination of genetics increased GDM prediction [28,29,30].

Lamri et al. found a polygenic risk score of GDM with a power of prediction of AUC = 0.62 using a cohort of South Asian women [28]. Kawai et al. described that the combination of clinical variables and a GRS improved the prediction of GDM with AUC = 0.70 among Caucasian women using the Carpentier and Coustan criteria for GDM [30]. Zulueta et al. carried out a case–control study of 139 Mexican women with GDM to build a GDM risk algorithm. They included four phenotypic variables, maternal age, pre-pregestational BMI, family history of T2DM, and previous pregnancies, as well as 11 SNPs, showing an AUC of 0.751 [31].

However, these results are not totally comparable since there are some inconsistencies across studies due to different sample sizes, populations, GDM screening, diagnostic criteria, and statistical analyses.

Regarding the candidate SNPs, we selected SNPs that are known to be associated with T2DM and/or GDM and previous SNPs explored in our cohort [22]. Therefore, the SNPs are located in genetic loci associated with glucose metabolism, such as rs1387153 and rs10830963 (melatonin receptor 1b MTNR1B), rs9368222 (cyclin-dependent kinase 5 regulatory subunit associated protein 1-like 1, CDKAL1), rs7651090 (insulin-like growth factor 2mRNA-binding protein 2, IGF2BP2), and rs1371614 (dihydropyrimidinase-related protein 5, DPYSL5).

These rs1387153 and rs10830963 polymorphisms of the MTN1RB gene are known to be associated with an increased risk of GDM [32]; an increased GDM risk in genetic models of the Chinese population, showing similar effect sizes [29]; and in genetic models of the Caucasian population with modest size effects [30]. In this regard, the rs10830963 variant in MTNR1B is associated with FPG values and impaired β-cell function [33] and might be considered a pharmacogenetic marker of antenatal insulin therapy in interactions with pre-pregnancy BMI [34]. rs1387153 is also related with an increase in FPG levels and T2DM prevalence in European populations [35]. The IGF2BP2 variant rs7651090 is described in Malaysian subjects with glutamic acid decarboxylase antibody (GADA)-negative diabetes [36]. rs1371614 DPYSL5 and rs9368222 CDKAL1 variants were previously reported in GDM women in our cohort [22].

On the contrary, we identified other SNPs that might confer a protective effect, such as the rs7607980 (cordon-bleu protein-like 1, COBLL1), rs10885122 (adrenoceptor α 2A, ADRA2A), rs1496653 (ubiquitin-conjugating enzyme E2, UBE2E2), rs340874 (proper homeobox protein-1, PROX1), and rs9368222 (CDK5 regulatory subunit associated protein 1 like 1, CDKAL1) variants. In line with this, rs7607980 is related to lower fasting insulin in children with overweight and obesity [37]. However, most variants have been reported to be associated with an increased risk of T2DM development.

Remarkably, we only found genetic variants of the MTNR1B gene in both races. No other SNPs were identified in common in CAU and LAT pregnant women.

To the best of our knowledge, this is the first study that examines significant associations between candidate SNPs, environmental factors, and GDM risk in genetic models in the Spanish population.

Our study exhibits several strengths. We studied our own cohort of subjects representing a broad spectrum of pregnant women. They were carefully characterized regarding phenotype, with clinical, demographic, and anthropometric data confirmed in a face-to-face appointment and with all patients performing our own OGTT, obtaining a GDM diagnosis based on validated diagnostic criteria.

On the contrary, there are some limitations that need to be acknowledged. Despite the important sample size of our study (1588 pregnant women), studies with larger sample sizes in different cohorts would be needed to validate this GRS model. Furthermore, a cost-effectiveness analysis might be crucial to implement it in clinical routine practice. In addition, this is an observational study, and we adjusted for multiple confounders; however, further underlying confounding factors might still be missed, which should be further considered: for instance, the effects of diet and physical activity. Finally, our study included only CAU and LAT women. Whether our findings can be generalized and replicated to other ethnic groups deserves further investigation.

## 5. Conclusions

In conclusion, adding genetic variants enhanced the prediction model of GDM risk in CAU and LAT pregnant women, facilitating a prompt identification and management of GDM. The present findings could entail modifications in our routine clinical practice. Further studies with larger sample sizes and longer follow-up are deemed relevant to further confirm our results.

## Figures and Tables

**Table 1:** Baseline characteristics at entry (<12 gestational weeks) of studied women by race and glucose tolerance groups.

	Caucasian Group				Latin American			
	Total (*n* = 1069)	GDM (*n* = 172)	NGT (*n* = 897)	*p*	Total (*n* = 519)	GDM (*n* = 83)	NGT (*n* = 436)	*p*
Age (years)	33 ± 4	34 ± 5	33 ± 4	0.083	30.9 ± 5.6	33 ± 5	30.6 ± 4	<0.001
Family history of T2DM	293 (27.4)	61 (35.5)	248 (27.6)	0.169	127 (24.5)	18 (21.7)	109 (25.0)	0.051
Family history of MetS	243 (22.7)	42 (24.4)	201 (22.4)	0.115	72 (13.9)	11 (13.3)	61 (14.0)	0.053
Previous history of GDM	40 (3.7)	12 (7.0)	28 (3.1)	0.017	16 (3.1)	9 (10.8)	7 (1.6)	<0.001
Previous history of Miscarriage	288 (26.9)	54 (31.4)	234 (26.1)	0.001	236 (45.5)	38 (45.8)	198 (45.4)	0.166
Primiparous	542 (50.7)	85 (49.4)	457 (50.9)	0.757	141 (27.2)	22 (26.5)	119 (27.3)	0.282
Pre-pregnancy BMI (kg/m^2^)	22.3 ± 3.4	23.6 ± 4.0	21.9 ± 3.2	<0.001	23.9 ± 3.7	25.0 ±3.8	23.6 ± 3.5	0.002
FPG (mg/dL)	80 ± 6	82 ± 6	79 ± 6	<0.001	80.4 ± 6.1	82 ± 6	80 ± 6	0.042
MEDAS score	5.0 ± 1.7	5.0 ± 1.6	5.0 ± 1.7	0.892	4.6 ± 1.8	4.7 ± 1.8	4.6 ± 1.8	0.529

Data are shown as mean ± SD or number (%). BMI, body mass index; GDM, gestational diabetes mellitus; NGT, normal glucose tolerance; MetS, metabolic syndrome; T2DM, type 2 diabetes mellitus; FPG, fasting plasma glucose; MEDAS Score: 14-point Mediterranean Diet Adherence Screener (MEDAS).

**Table 2:** ROC analysis for the logistic regression for Caucasian women.

	AUC	Threshold	Sensitivity	Specificity	PPV	NPV
Age	0.535	0.167	0.4207	0.6491	0.187	0.8539
Age + Pre-pregnancy BMI	0.574	0.199	0.2561	0.8538	0.2516	0.857
Age + Pre-Pregnancy BMI + FPG 12 GW	0.644	0.180	0.561	0.6795	0.250	0.8892
Age + Pre-pregnancy BMI + rs10830963 + rs7651090 + rs180587 + rs7607980 + rs1371614 + rs3783347	0.684	0.137	0.7439	0.5333	0.2346	0.9162
Age + Pre-pregnancy BMI + FPG 12 GW+ rs10830963 + rs180587 + rs7607980 + rs7651090 + rs1371614	0.714	0.159	0.6646	0.6901	0.2919	0.9149

BMI, body mass index; FPG, fasting plasma glucose; GW, gestational weeks.

**Table 3:** ROC analysis of the logistic regression for Latin American women.

	AUC	Threshold	Sensitivity	Specificity	PPV	NPV
Age	0.543	0.182	0.7494	0.7916	0.217	0.8521
Age + pre-pregnancy BMI	0.651	0.134	0.6914	0.5831	0.2448	0.9071
Age + pre-pregnancy BMI + FPG 12 GW	0.685	0.133	0.7975	0.4771	0.225	0.9254
Age + pre-pregnancy BMI + rs10830963 + rs7651090 + rs7607980 + rs1371614 + rs180587 + rs3783347	0.745	0.156	0.6914	0.5831	0.2446	0.9065
Age + pre-pregnancy BMI + FPG 12 GW + rs10885122 + rs1496653 + rs340874 + rs7041847 + rs9368222 + rs1387153	0.760	0.195	0.6203	0.8072	0.3792	0.9174

BMI, body mass index; FPG, fasting plasma glucose; GW, gestational weeks.

## Data Availability

The data analysed in this study are subject to the following licenses/restrictions: no restriction. Requests to access these datasets should be directed to acalle.edu@gmail.com.

## Supplementary Materials

The following supporting information can be downloaded at https://www.mdpi.com/article/10.3390/genes15040482/s1, Table S1: The SNP considered for CAU women; Table S2: The SNP considered for LAT women; Table S3: Logistic regression model for CAU women including age and pre-pregnancy BMI; Table S4: Logistic regression model also considering FPG 12 GW for CAU women; Table S5: Logistic regression model for LAT women, including age and pre-pregnancy BMI; Table S6: Logistic regression model also considering FPG 12 GW for LAT women; Figure S1: ROC for CAU, Panel (a) for age. Panel (b) for age and BMI. Panel (c) for age, BMI, and FPG. Panel (d) for relevant snips and age, BMI, and FPG; Figure S2: ROC for LAT. Panel (a) for age. Panel (b) for age and BMI. Panel (c) for age, BMI, and FPG. Panel (d) for relevant snips and age, BMI, and FPG.
