# Supplementary material for: A Simplified Screening Model to Predict the Risk of Gestational Diabetes Mellitus in Caucasian and Latin American Pregnant Women
Source: Genes (Basel). 2024 Apr 11;15(4):482. doi: 10.3390/genes15040482 (PMC11049498; doi:10.3390/genes15040482)
Supplement: Supplementary file 1 [file genes-15-00482-s001.zip › genes-2926607-supplementary.pdf]

Title: A Simplified Screening Model to Predict the Risk of Gestational Diabetes Mellitus in Caucasian and Latin American Pregnant Women

Maria Arnoriaga-Rodriguez et al

Table S1. The SNPs considered for CAU women

		Gestational diabetes mellitus		
		NO	YES	p
rs10830962	REF – CC	292 (34.2)	43 (26.2)	0.0476
	ALT – CG+GG	563 (65.8)	121 (73.8)	
rs1387153	REF – CC	447 (52.3)	68 (41.5)	0.0111
	ALT – CT+TT	408 (47.7)	96 (58.5)	
rs180587	REF – AA	559 (65.4)	124 (75.6)	0.0107
	ALT – AG+GG	296 (34.6)	40 (24.4)	
rs3746750	REF – AA	95 (11.1)	13 (7.9)	0.225
	ALT – AG+GG	760 (88.9)	151 (92.1)	
rs780094	REF – TT	211 (24.7)	28 (17.1)	0.0352
	ALT – TC+CC	644 (75.3)	136 (82.9)	
rs4402960	REF – GG	404 (47.3)	57 (34.8)	0.00323
	ALT – GT+TT	451 (57.7)	107 (65.2)	
rs10814916	REF – AA	191 (22.3)	42 (25.6)	0.361
	ALT – AC+CC	664 (77.7)	122 (74.4)	
rs10830963	REF – CC	476 (55.7)	69 (42.1)	0.00138
	ALT – CG+GG	379 (44.3)	95 (57.9)	
rs1496653	REF – AA	573 (67.0)	114 (69.5)	0.532
	ALT – AG+GG	282 (33.0)	50 (30.5)	
rs17676067	REF – TT	466 (54.5)	80 (48.8)	0.178
	ALT – TC+CC	389 (45.5)	84 (51.2)	
rs5215	REF – CC	102 (11.9)	22 (13.4)	0.594
	ALT – CT+TT	753 (88.1)	142 (86.6)	
rs6048205	REF – AA	771 (90.2)	155 (94.5)	0.0773
	ALT – AG+GG	84 (9.8)	9 (5.5)	
rs7041847	REF – AA	224 (26.2)	55 (33.5)	0.0536
	ALT – AG+GG	631 (73.8)	109 (66.5)	
rs7607980	REF – TT	613 (71.7)	132 (80.5)	0.02
	ALT – TC+CC	242 (28.3)	32 (19.5)	
rs7651090	REF – AA	402 (47.0)	56 (34.1)	0.0024
	ALT – AG+GG	453 (53.0)	108 (65.9)	
rs10747083	REF – GG	95 (11.1)	26 (15.9)	0.0855
	ALT – GA+AA	760 (88.9)	138 (84.1)	
rs11671664	REF – GG	661 (77.3)	112 (68.3)	0.0134
	ALT – GA+AA	194 (22.7)	52 (31.7)	
rs1371614	REF – CC	490 (57.3)	76 (46.3)	0.00962
	ALT – CT+TT	365 (42.7)	88 (53.7)	
rs2293941	REF – GG	510 (59.6)	85 (51.8)	0.0627
	ALT – GA+AA	345 (40.4)	79 (48.2)	
rs3783347	REF – GG	569 (66.5)	122 (74.4)	0.049
	ALT – GT+TT	286 (33.5)	42 (25.6)	

Differences in genetic variants between Caucasian pregnant women with and without GDM.
SNPs, single nucleotide polymorphisms, LAT, Latin American

Table S2. The SNP considered for LAT women

		Gestational diabetes mellitus		
		NO	YES	
		N (%)	N (%)	P
rs1387153	REF – CC	291 (70.1)	49 (60.5)	0.0879
	ALT – CT+TT	124 (29.9)	32 (39.5)	
rs563694	REF – CC	7 (1.7)	2 (2.5)	0.6290
	ALT – CA+AA	408 (98.3)	79 (97.5)	
rs737288	REF - GG	257 (61.9)	51 (63.0)	0.8610
	ALT – GT+TT	158 (38.1)	30 (37.0)	
rs780094	REF - TT	43 (10.4)	10 (12.3)	0.5970
	ALT – TC+CC	372 (89.6)	71 (87.7)	
rs1496653	REF – AA	322 (77.6)	70 (86.4)	0.0742
	ALT – AG+GG	93 (22.4)	11 (13.6)	
rs340874	REF – TT	162 (39.0)	44 (54.3)	0.0107
	ALT – TC+CC	253 (61.0)	37 (45.7)	
rs7041847	REF – AA	133 (32.0)	37 (45.7)	0.0181
	ALT – AG+GG	282 (68.0)	44 (54.3)	
rs10747083	REF – GG	24 (5.8)	6 (7.4)	0.5750
	ALT – GA+AA	391 (94.2)	75 (92.6)	
rs2302593	REF – CC	74 (17.8)	20 (24.7)	0.1500
	ALT – CG+GG	341 (82.2)	61 (75.3)	
rs2943634	REF – AA	31 (7.5)	4 (4.9)	0.4160
	ALT – AC+CC	384 (92.5)	77 (95.1)	
rs9368222	REF – CC	258 (62.2)	38 (46.9)	0.0105
	ALT – CA+AA	157 (37.8)	43 (53.1)	
rs10885122	REF – TT	14 (3.4)	8 (9.9)	0.0093
	ALT – TG+GG	401 (96.6)	73 (90.1)	
rs11605924	REF – AA	100 (24.1)	23 (28.4)	0.4130
	ALT – AC+CC	315 (75.9)	58 (71.6)	
rs2293941	REF – GG	222 (53.5)	34 (42.0)	0.0578
	ALT – GA+AA	193 (46.5)	47 (58.0)	

Differences in genetic variants between Latin American pregnant women with and without GDM.
SNPs, single nucleotide polymorphisms, CAU,
Caucasian

Table S3. Logistic regression model for CAU women including age and pre-pregnancy BMI

	OR	95%CI		P
Intercept	0.08906893	0.05552965	0.1395095	<0.001
Age: > 35	1.46702067	1.02831632	2.0871113	0.03356
Pre-BMI: >=25	2.12206154	1.39269919	3.1954808	0.000372
rs10830963: ALT	1.80737864	1.27820687	2.5680312	0.000868
rs7651090: ALT	1.72787979	1.21179796	2.4865168	0.002813
rs7607980: ALT	0.57979453	0.3733052	0.8777595	0.012205
rs1371614: ALT	1.66053987	1.17503854	2.3533848	0.004155
rs180587: ALT	0.59469219	0.39711646	0.8745811	0.009704
rs3783347: ALT	0.66739288	0.44805113	0.9779226	0.041829

Pre-BMI, pre-pregnancy body mass index. CAU, Caucasian

Table S4. Logistic regression model also considering FPG 12 GW for CAU women

	OR	95%CI		P
Intercept	0.05549941	0.03354322	0.08923539	0.03219
Age: > 35	1.47994495	1.03212137	2.11709263	0.00287
Pre-BMI: >=25	1.90343168	1.23794349	2.89063533	<0.001
FPG 12 GW: > 83.5	2.62071031	1.84036658	3.73846355	0.00122
rs10830963: ALT	1.79095409	1.26036053	2.55697023	0.00364
rs7651090: ALT	1.71505434	1.19687989	2.47993587	0.00625
rs180587: ALT	0.57279023	0.38036776	0.84703779	0.00371
rs1371614: ALT	1.68456862	1.18580673	2.40079062	0.01396
rs7607980: ALT	0.58269177	0.37360424	0.88613394	0.03219

FPG, Fasting plasma glucose; Pre-BMI, pre-pregnancy body mass index. CAU, Caucasian

Table S5. Logistic regression model for LAT women including age and pre-pregnancy BMI

	OR	95%CI		P
Intercept	0.5497175	0.1786628	1.6171092	0.28288
Age: > 35	2.1312221	1.1845606	3.7870795	0.0104
Pre-BMI: >=25	2.8542084	1.6995756	4.8106824	<0.001
rs7041847: ALT	0.4852078	0.2864382	0.8211108	0.00694
rs340874: ALT	0.4974073	0.2952994	0.8313401	0.00799
rs9368222: ALT	1.7668956	1.0619215	2.9517288	0.02861
rs10885122: ALT	0.2966123	0.1100393	0.8418865	0.0179
rs1496653: ALT	0.4581395	0.2124372	0.9080521	0.03376
rs1387153: ALT	1.8760245	1.0922772	3.2094297	0.02171

Pre-BMI, pre-pregnancy body mass index. LAT, Latin American

Table S6. Logistic regression model also considering FPG 12 GW for LAT women

	OR	95%CI		P
Intercept	0.4723906	0.1497954	1.4159917	0.186671
Age: > 35	1.9855988	1.0866697	3.5801662	0.023587
Pre-BMI: [25, 30)	2.627349	1.5426087	4.482802	0.000371
FPG 12 GW: > 82.5	2.0526991	1.2143135	3.4922313	0.007435
rs340874: ALT	0.4870384	0.2853661	0.8245569	0.007682
rs10885122: ALT	0.2562977	0.0946125	0.7344631	0.008427
rs9368222: ALT	1.8526053	1.0997165	3.1374001	0.020757
rs1496653: ALT	0.3823132	0.1644848	0.7974975	0.015927
rs7041847: ALT	0.4918504	0.2873367	0.8413493	0.009405
rs1387153: ALT	1.8471792	1.0601884	3.2048885	0.029122

FPG, Fasting plasma glucose; Pre-BMI, pre-pregnancy body mass index. LAT, Latin American

Figure S1. ROC for CAU
Panel a for age

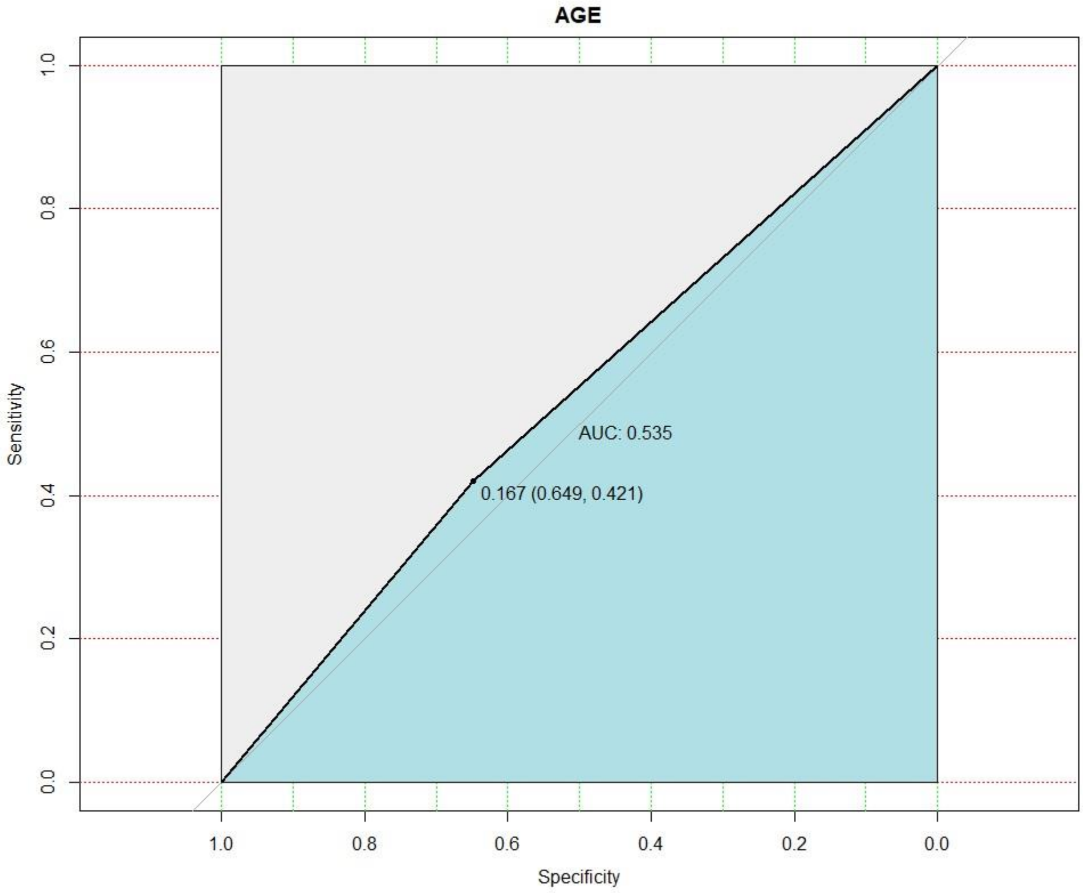

Panel b for age and BMI

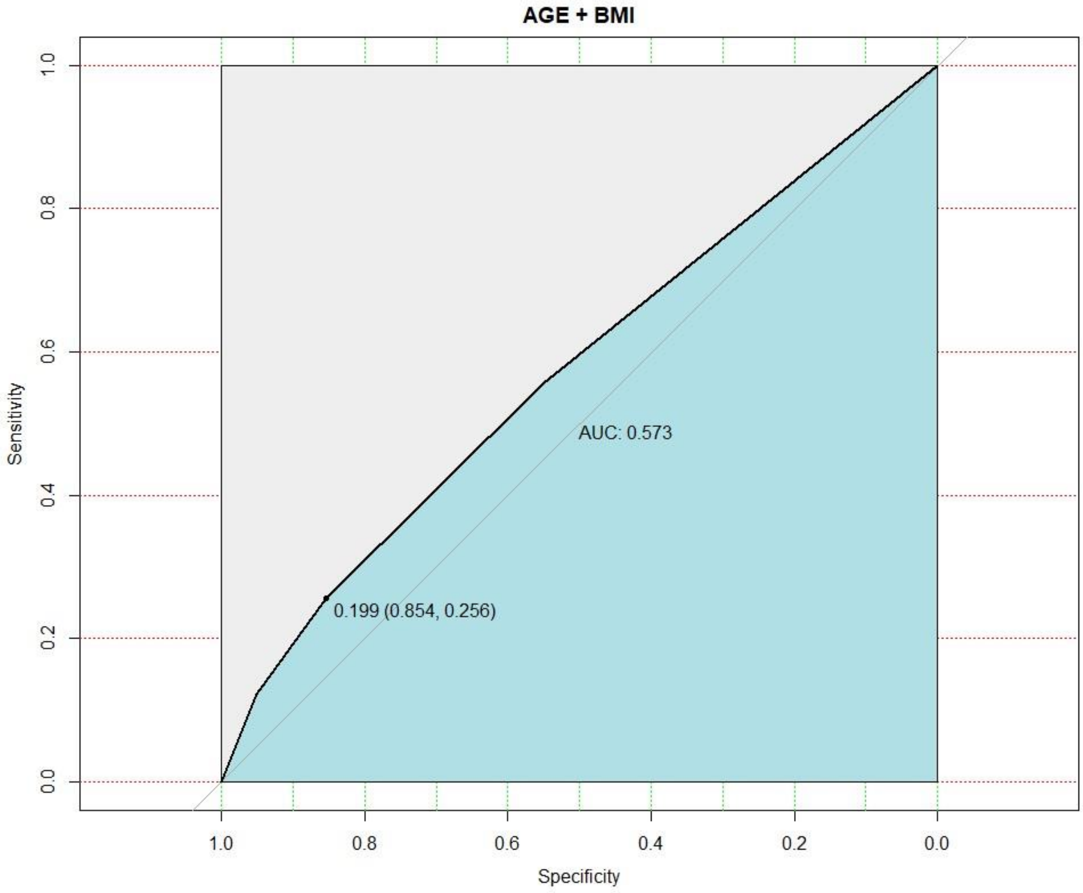

Panel C for Age, BMI and FPG

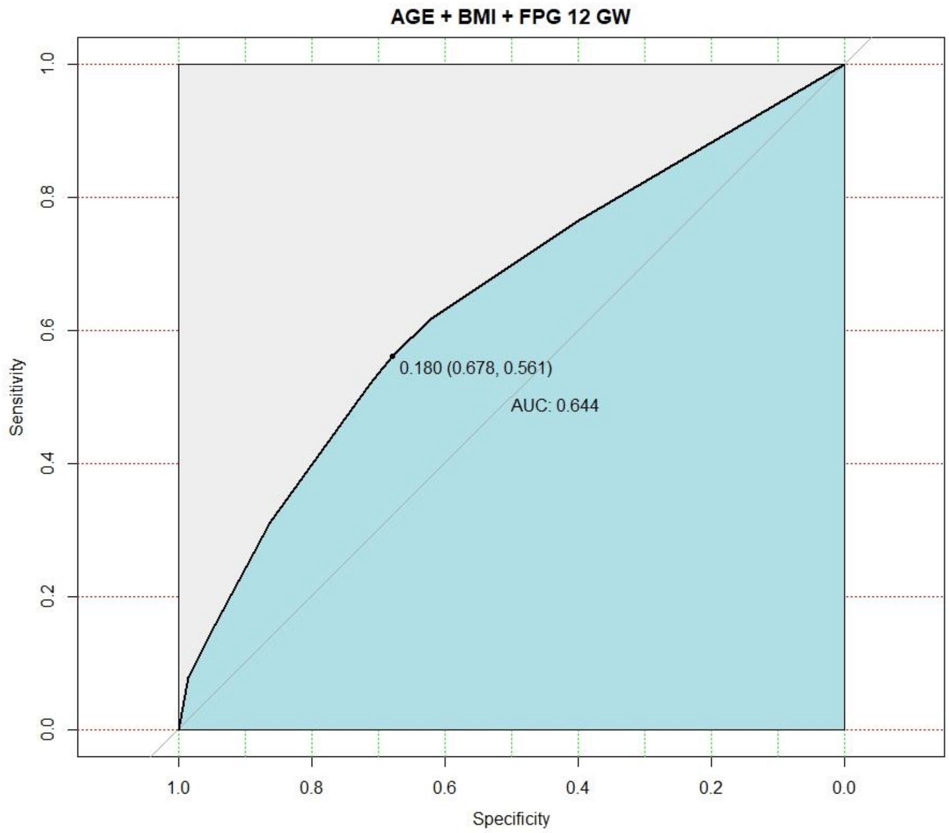

Panel d For Relevant SNPs and age, BMI and FPG

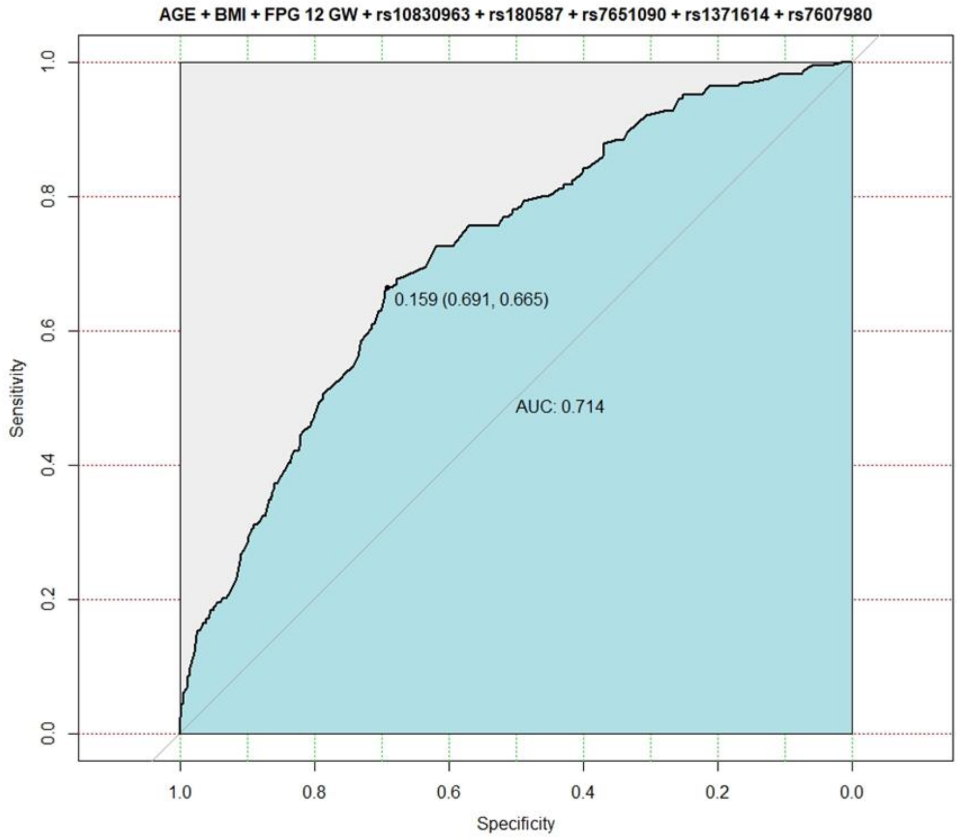

CAU, Caucasian; FPG, fasting plasma glucose; GW, gestational week.

Figure S2 ROC for LAT
Panel a for Age

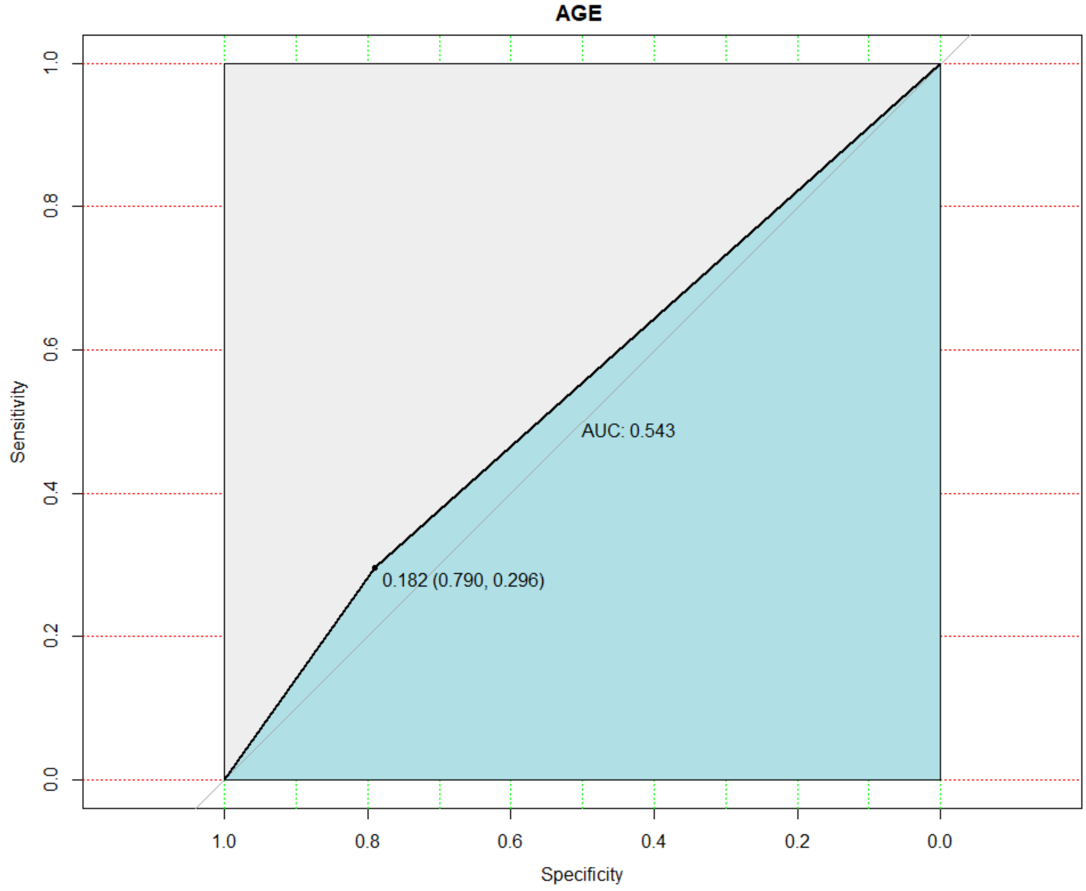

Panel b for Age and BMI

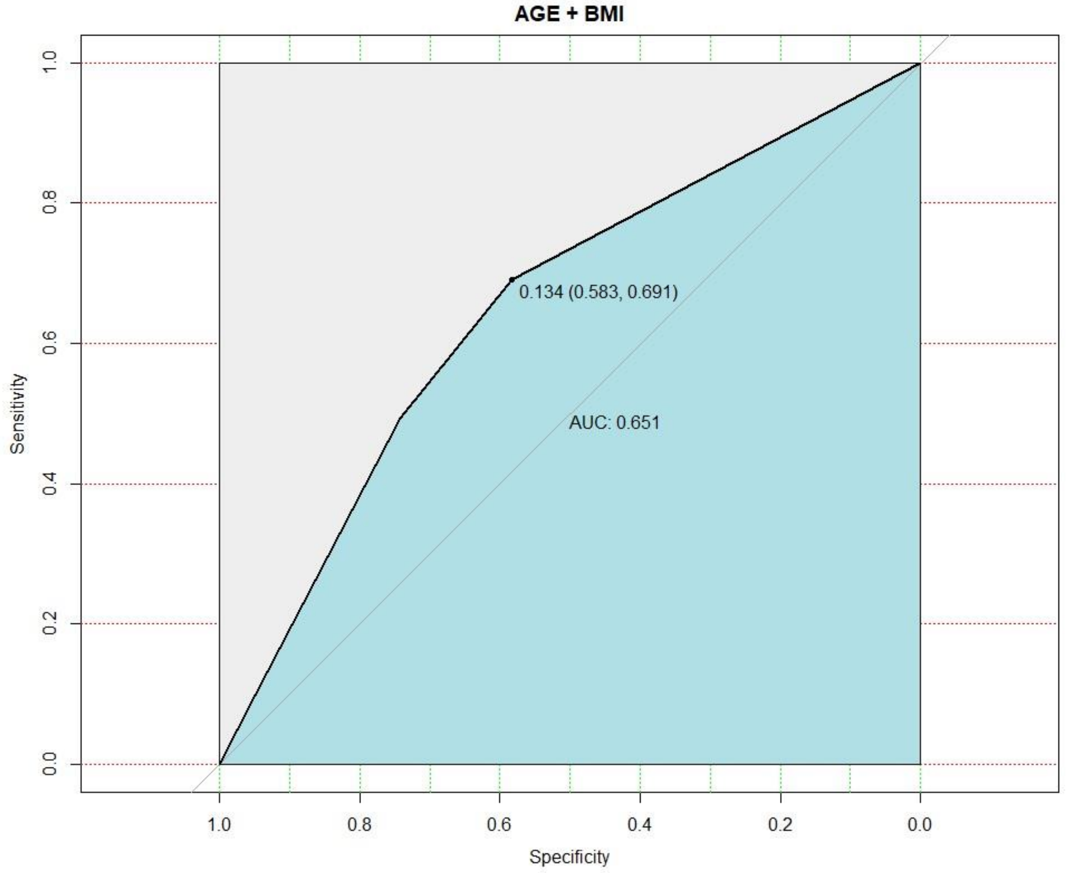

Panel c for Age, BMI and FPG

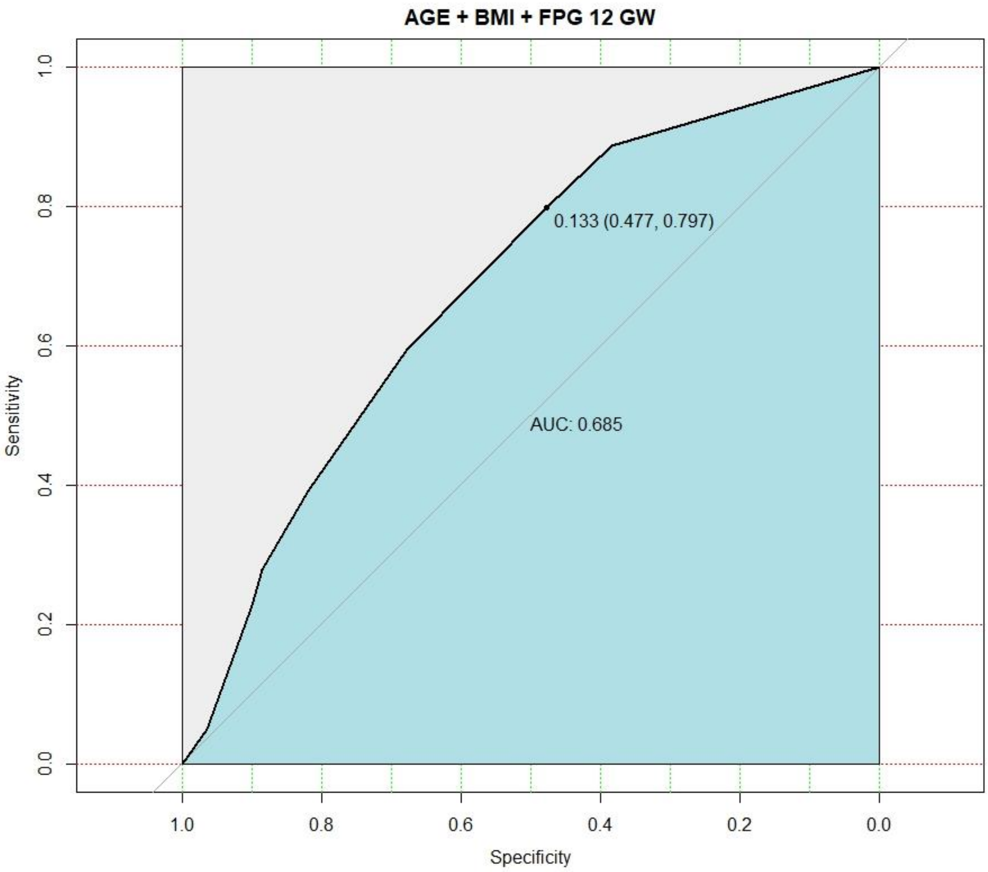

Panel d For Relevant SNPs and age, BMI and FPG

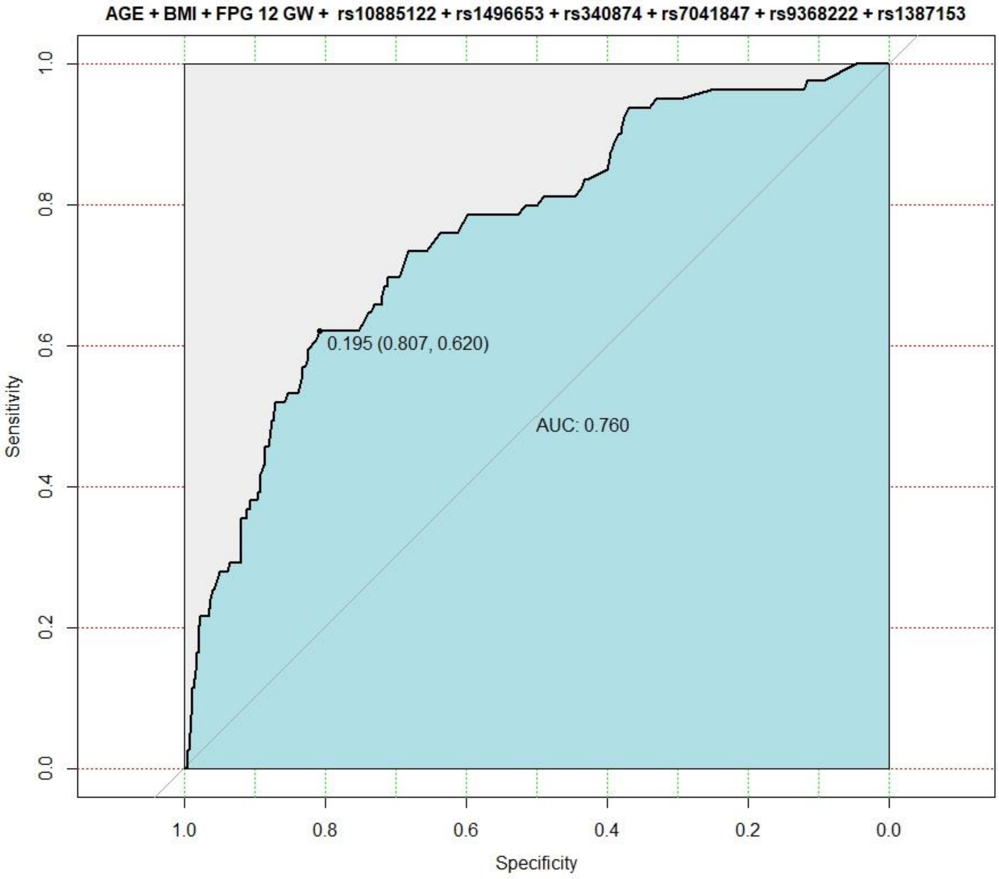

LAT,
Latin American; FPG, fasting plasma glucose; GW, gestational week.
